# Short Expandable-Wing Suture Anchor for Osteoporotic and Small Bone Fixation: Validation in a 3D-Printed Coracoclavicular Reconstruction Model

**DOI:** 10.3390/jfb16100379

**Published:** 2025-10-10

**Authors:** Chia-Hung Tsai, Shao-Fu Huang, Rong-Chen Lin, Pao-Wei Lee, Cheng-Ying Lee, Chun-Li Lin

**Affiliations:** 1 Department of Biomedical Engineering, National Yang Ming Chaio Tung University, Taipei 112, Taiwan; cckerer@gmail.com (C.-H.T.); shaofu.h@gmail.com (S.-F.H.); rclin1126.be12@nycu.edu.tw (R.-C.L.); paoweilee.be11@nycu.edu.tw (P.-W.L.); lcying.be13@nycu.edu.com (C.-Y.L.); 2 Department of Orthopedics, Tri-Service General Hospital Songshan Branch, National Defense Medical University, Taipei 112, Taiwan; 3 Medical Device Innovation & Translation Center, National Yang Ming Chiao Tung University, Taipei 112, Taiwan

**Keywords:** suture anchor, osteoporotic, coracoclavicular, 3D-printing, pull-out

## Abstract

Suture anchors are widely used for tendon and ligament repair, but their fixation strength is compromised in osteoporotic bone and limited bone volume such as the coracoid process. Existing designs are prone to penetration and insufficient cortical engagement under such conditions. In this study, we developed a novel short expandable-wing (SEW) suture anchor (Ti6Al4V) designed to enhance pull-out resistance through a deployable wing mechanism that locks directly against the cortical bone. Finite element analysis based on CT-derived bone material properties demonstrated reduced intra-bone displacement and improved load transfer with the SEW compared to conventional anchors. Mechanical testing using matched artificial bone surrogates (*N* = 3 per group) demonstrated significantly higher static pull-out strength in both normal (581 N) and osteoporotic bone (377 N) relative to controls (*p* < 0.05). Although the sample size was limited, results were consistent and statistically significant. After cyclic loading, SEW anchor fixation strength increased by 25–56%. In a 3D-printed anatomical coracoclavicular ligament reconstruction model, the SEW anchor provided nearly double the fixation strength of the hook plate, underscoring its superior stability under high-demand clinical conditions. This straightforward implantation protocol—requiring only a 5 mm drill hole without tapping, followed by direct insertion and knob-driven wing deployment—facilitates seamless integration into existing surgical workflows. Overall, the SEW anchor addresses key limitations of existing anchor designs in small bone volume and osteoporotic environments, demonstrating strong potential for clinical translation.

## 1. Introduction

Suture anchors are widely used in ligament and tendon repair, effectively securing soft tissue to bone surfaces, and are important implants for shoulder and other joint reconstructions [1,2,3,4,5,6,7]. Commonly used clinical designs include threaded, impaction, and all-suture anchors, with materials ranging from titanium metal and polyether ether ketone (PEEK) to biodegradable composites. Titanium screw anchors are favored for their excellent biocompatibility, mechanical properties, and intramedullary fixation effect. However, existing designs still have limitations in specific clinical situations, particularly in areas with osteoporosis and restricted bone size, where initial fixation stability and safety may be insufficient [1,8,9,10,11,12,13,14].

Patients with osteoporosis experience a significant decline in the mechanical strength of cancellous bone, leading to increased loosening of suture anchors under load, higher expulsion rates, and postoperative failure rates of up to 22.8% [15,16]. Although cortical bone thickness decreases with the progression of osteoporosis, the reduction in its mechanical strength is relatively limited, and it remains crucial for maintaining overall structural stability [17,18,19,20]. To address these challenges, the literature indicates that increasing thread density can enhance the contact area between the anchor and cortical bone, thereby improving initial fixation strength and pull-out resistance [21]. Additionally, a deployable wing structure post-implantation can create a mechanical barrier within cortical bone, not only enhancing pull-out resistance but also generating localized compressive stress to promote bone formation [1,14,22,23,24]. On the other hand, if osteoporosis occurs in small-sized bone areas such as the coracoid process, existing anchors with large-pitch designs reduce the contact area with cortical bone, further limiting initial fixation strength. Moreover, the length of current commercial anchors, often 14–18 mm, can easily penetrate the bone and weaken fixation. However, most existing anchors do not simultaneously consider shortening the anchor length to adapt to small-sized bone areas while also improving initial stability under osteoporotic conditions [10].

Nevertheless, all-suture anchors, while specifically designed for small bone volumes, remain limited by their dependence on cortical thickness, susceptibility to tunnel widening, and relatively low pull-out strength under high-load applications such as coracoclavicular ligament reconstruction [5,16,25,26,27,28,29]. These limitations highlight the need for anchors that combine small dimensions with robust cortical engagement.

This study presents a novel suture anchor with a short and winged deployment mechanism. The winged structure expands within cancellous bone after implantation and advances upward to engage the cortical bone, forming a mechanical locking effect that enhances pull-out resistance. The design length was reduced to ≤10 mm to accommodate areas with limited bone size, and it was manufactured using highly biocompatible Ti6Al4V titanium alloy. To ensure the reliability and clinical feasibility of this design, finite element (FE) analysis was conducted to simulate stress distribution and pull-out behavior under different bone quality conditions. Subsequently, static pull-out tests and pull-out tests after dynamic loading were performed on standardized artificial bone models to evaluate initial stability and durability.

After completing mechanical validation, a coracoclavicular (CC) ligament reconstruction model was employed to evaluate the performance of the novel anchor under high-demand clinical conditions. The CC ligament is a key structure for acromioclavicular joint stability; however, its anatomical site is characterized by limited bone dimensions and a high proportion of cortical load-bearing, which impose stringent requirements on anchor fixation [30,31,32,33]. The current fixation methods of CC ligament reconstruction are associated with notable complications. Hook plates, although effective for reduction, can cause subacromial impingement, hardware irritation, and often require removal surgery [30,31,32]. Conventional screw anchors may loosen in osteoporotic or small bones, while all-suture anchors risk tunnel enlargement and loss of fixation. Clinical studies report recurrent instability, hardware migration, and reoperation as common complications, emphasizing the unmet need for more reliable fixation options [33,34,35,36,37,38,39].

In this study, two novel short expandable wing (SEW) anchors were implanted into the coracoid process, with sutures passed through the clavicle, and two impaction-type anchors were used to secure the sutures to the clavicle. Their biomechanical pull-out strength was then compared with that of the conventional hook plate for clavicle–scapula fixation. We hypothesize that this new design can provide superior stability in the constrained cortical bone environment, offering a better alternative for clinical acromioclavicular joint reconstruction. Looking forward, further cadaveric validation is needed to account for heterogeneous bone microstructures, along with long-term cyclic and off-axis loading tests to replicate physiological conditions. Ultimately, clinical studies will be required to confirm the safety, durability, and translational potential of the SEW anchor in patients with osteoporotic and small-dimension bones.

## 2. Materials and Methods

### 2.1. Design Concept of SEW Suture Anchor & Instrument

The design concept was motivated by the clinical challenges of fixation in osteoporotic and small-dimension bones, such as the coracoid process, where conventional screw anchors risk loosening, cortical penetration, or insufficient fixation strength. To address this, the SEW anchor was designed in a short configuration to minimize the risk of cortical breach, while incorporating deployable wings that expand internally to lock against the cortical surface and enhance pull-out resistance [12].

**Figure 1:**
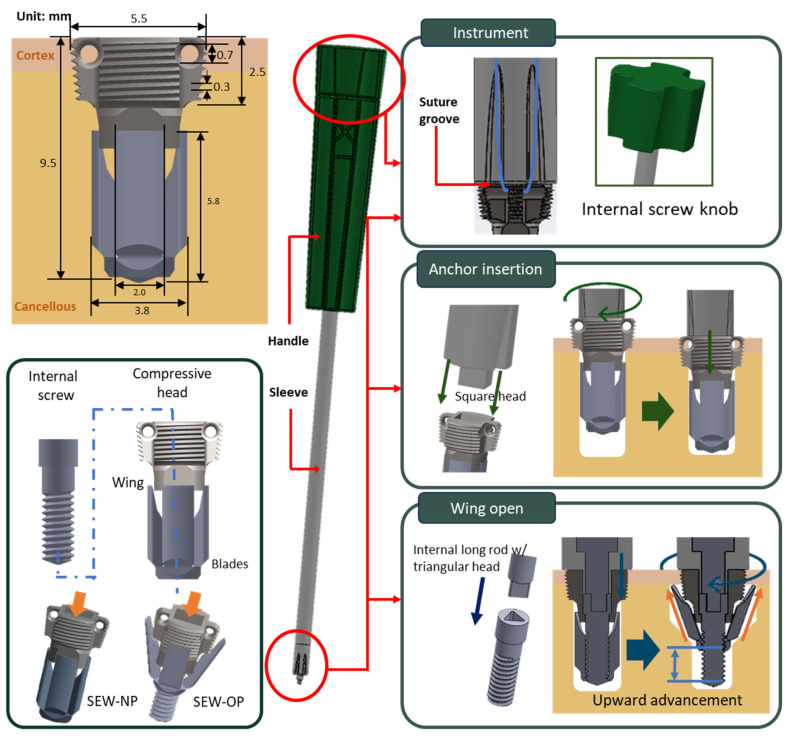
Design of the SEW anchor and instrument. Top left: dimensional drawing of the SEW anchor; bottom left: components including compressive head, four-blade body, and interlocking internal screw; center: instrument schematic; top right: knob and sleeve head grooves; middle right: square head of the sleeve with operation schematic; bottom right: triangular head of the internal screw with operation schematic.

The anchor adopted a short structure (total length 9.5 mm) with 5.5 mm in diameter to reduce the risk of penetrating bone in small-sized bones such as the coracoid process. The body was composed of two main structures: the compressive head and the wing, which interlocked through a central internal screw. The external threads of the head adopted a dual-thread design (pitch 0.3 mm) with a 7° pressure angle, which increased the contact area and radial pressure with the cortical bone, enhancing initial insertion strength and pull-out resistance. The threaded head had suture holes with a diameter of 0.7 mm on both sides, with rounded edges to reduce suture friction damage; after the suture passed through the holes, it could be temporarily fixed in the suture groove of the corresponding instrument handle to avoid tangling or knotting during the insertion process (Figure 1).

The wing section was composed of four blades, which were driven to expand by a central internal screw embedded in the head. When the internal screw rotated, the wings unfolded outward along the guiding slope of the head and advanced upward, eventually pressing against the inner edge of the cortical bone, forming a barbed mechanical block that significantly enhanced pull-out resistance (Figure 1). Under normal bone conditions, the wing upward advancement was approximately 1.9 mm, while under osteoporotic conditions, it was about 3.0 mm. This structure not only provided stable initial fixation under osteoporotic conditions, but the radial compressive stress applied to the surrounding bone when the wings were deployed could also stimulate local osteogenesis, aiding in long-term fixation stability and bone integration.

The instrument consisted of an external handle, a sleeve, and a square head at the front, which connected to an internal long rod, a plastic knob at the back, and a triangular head at the front. The front end of the sleeve was equipped with a wire groove and a wire channel, which secured and protected the suture during the operation, preventing it from tangling or wearing out when being screwed in. During the implantation phase, the square head precisely fit into the square hole at the head of the anchor, and the anchor was inserted into the bone by rotating the handle. During the wing deployment phase, the knob drove the triangular head, which in turn rotated the internal screw of the anchor, causing the wings to unfold outward along the guiding slope and advance upward, securing themselves to the inner edge of the cortical bone to enhance pull-out resistance (Figure 1).

### 2.2. HU-Based Mechanical Property Conversion and Artificial Bone Matching

The proximal humerus (PH) was the common site for anchor implantation. To ensure the consistency of bone material properties in subsequent FE analysis and mechanical testing, the bone condition was quantitatively assessed through the Hounsfield unit (HU) values measured by clinical computed tomography (CT) imaging [17,18,19,20]. According to the study by Liu et al., 2025, patients were grouped based on the lowest T-score (femoral neck or lumbar spine) obtained using dual-energy X-ray absorptiometry (DXA) according to the World Health Organization (WHO) bone density diagnostic standards [19]. The normal bone group (T-score ≥ −1) PH average HU value was 90.79 ± 30.32, reflecting healthy bone quality and intact trabecular structure; the osteoporotic bone group (T-score ≤ −2.5) PH average HU value was 35.07 ± 18.42, indicating a significant decrease in trabecular bone density and structure. To convert HU values into material parameters that could reflect mechanical behavior, this study adopted the conversion formula between HU and bone mineral density (BMD, g/cc) established by Reeves et al., 2018 [20]:

BMD = 6.295 × 10^−4^ × HU + 4.449 × 10^−2^

And the conversion formula between BMD and Young’s modulus (E, MPa) proposed by Morgan et al., 2003 [18] was also used:

E = 4730 × BMD^1.56^

Based on this calculation, the BMD of normal trabecular bone (HU = 90.79) was 0.102 g/cc, with an elastic modulus (E) of approximately 133.63 MPa; the BMD of osteoporotic bone (HU = 35.07) was 0.067 g/cc, with an elastic modulus of approximately 69.05 MPa. This conversion process directly transformed CT imaging data into material properties for finite element analysis.

**Figure 2:**
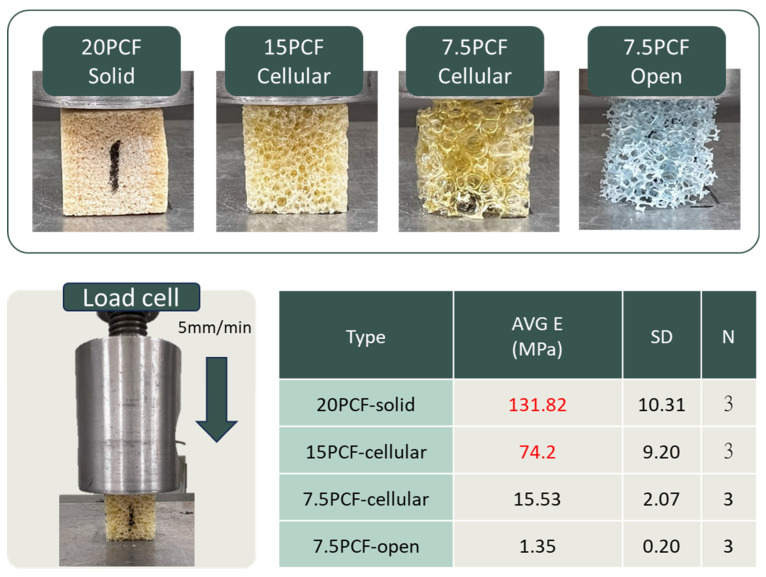
Compression testing of artificial bone blocks with different densities. Representative images of 20 PCF solid, 15 PCF cellular, 7.5 PCF cellular, and 7.5 PCF open foam blocks (**top**). Schematic of compression setup with a load cell at 5 mm/min (**bottom left**). Average elastic modulus (E), standard deviation (SD), and sample number (*N* = 3) for each group are summarized in the table (**bottom right**).

To establish the consistency of subsequent mechanical testing experiments, this study tested various densities and structural forms of artificial bone (Sawbones, Vashon Island, WA, USA), including 20 PCF solid, 15 PCF cellular, 7.5 PCF cellular, and 7.5 PCF open types. The elastic modulus was obtained through compression tests (15 × 15 × 15 mm^3^, loading speed 5 mm/min) and compared with the calculated E values (Figure 2). Ultimately, 20 PCF Solid (E ≈ 131.82 MPa) was selected as the corresponding material for normal bone quality, and 15 PCF Cellular (E ≈ 74.20 MPa) was chosen as the corresponding material for osteoporotic bone quality. This pairing method ensured that the mechanical properties of the experimental bone blocks were similar to the clinical proximal humeral bone quality, thereby enhancing the clinical relevance of the test results.

### 2.3. Finite Element Analysis for SEW Suture Anchor Under Different Bone Quality

**Figure 3:**
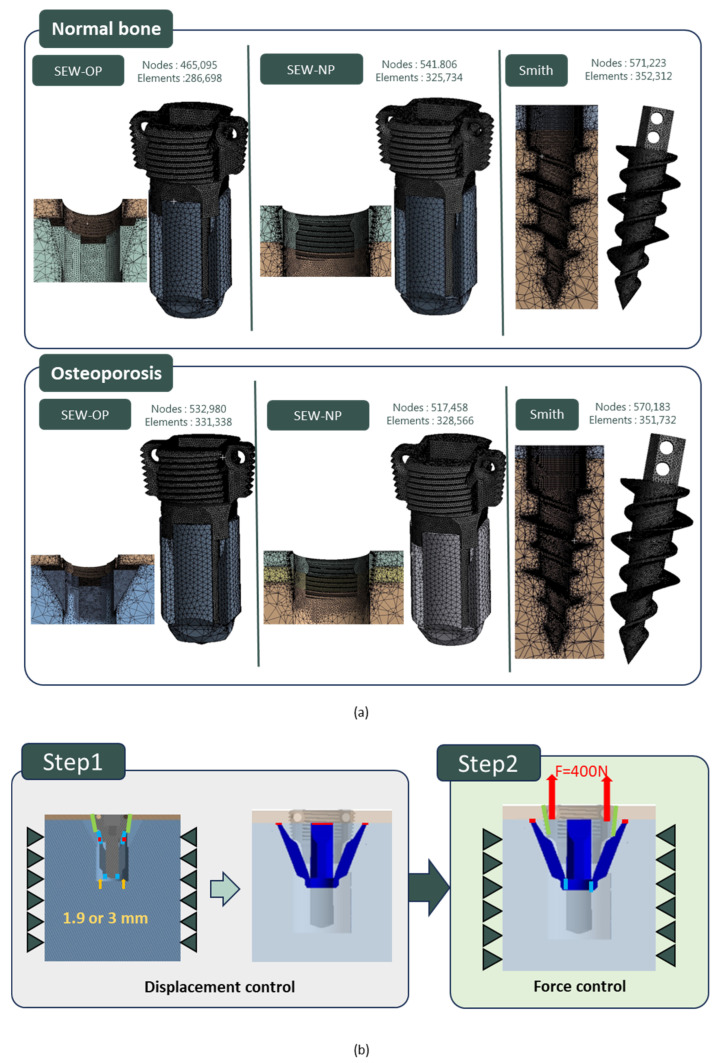
Finite element analysis (FEA) setup for SEW and control anchors. (**a**) Mesh models of SEW-OP, SEW-NP, and Smith anchors in normal and osteoporotic bone conditions, showing corresponding node and element counts. (**b**) Two-stage simulation process: Step 1—displacement control for wing expansion (1.9 mm in normal bone or 3.0 mm in osteoporotic bone); Step 2—force control with an upward pull-out load of 400 N applied to the anchor head.

The mechanical performance and stress distribution of the new SEW anchor in normal and osteoporotic bones were evaluated using FE analysis. CAD models, including SEW-NP (SEW anchor without wing opening), SEW-OP (SEW anchor with wing opening), and the control group Smith (diameter 5.5 mm and length 16 mm; TWINFIX Ultra HA anchor, Smith & Nephew, Andover, MA, USA), were established and imported into the FE software ANSYS Workbench 2024R2, and corresponding bone models (length and width 20 mm) were created. These included a cortical bone thickness of 2 mm for the normal group, 1 mm for the osteoporotic group, and a trabecular bone thickness of 30 mm for both groups. The material parameters were set to include titanium alloy (ASTM F136 [39]), cortical bone (Sawbones standard values), and trabecular bone elastic moduli of 140 MPa (normal) and 70 MPa (osteoporotic), respectively. The mesh used tetrahedral elements, with a mesh size of 0.1 mm for the critical contact area (bone–thread), 0.2 mm for the wing area, and 1.5 mm for the remaining bone areas (Figure 3a). The displacement of the nodes around the bone block was set to zero as the boundary condition.

In all analyses, the bone–anchor interface was simulated using contact elements, with a friction coefficient of μ = 0.6 for the normal group and μ = 0.3 for the osteoporotic group. In the SEW-NP and Smith groups, a single-stage analysis was conducted, directly applying a 400 N upward force to the anchor to simulate pull-out. In the SEW-OP group, a two-stage simulation was used. In the first stage, displacement control was employed, setting the wings to open and slide upward (1.9 mm for the normal group and 3.0 mm for the osteoporotic group). The contact coefficient between the wings and the head was set to 0.1, causing compression with the cortical bone. In the second stage, based on the results of the first stage, a 400 N upward force was applied to the head of the anchor to simulate pull-out (Figure 3b). The analysis results compared the stress patterns and displacement responses of the three models under different bone quality conditions, and these results were then compared with subsequent experimental findings.

### 2.4. Manufacturing of SEW Suture Anchor and Static/Dynamic Mechanical Testing

This study employed CNC turning–milling hybrid processing to manufacture the anchors, simultaneously considering tool dimensions and machining path constraints during the design phase to ensure precision and efficiency. The material selected was the biocompatible Ti6Al4V titanium alloy. The handle and knob of the instrument were produced by injection molding and combined with the sleeves, square heads, inner rods, and triangular heads through milling processes.

**Figure 4:**
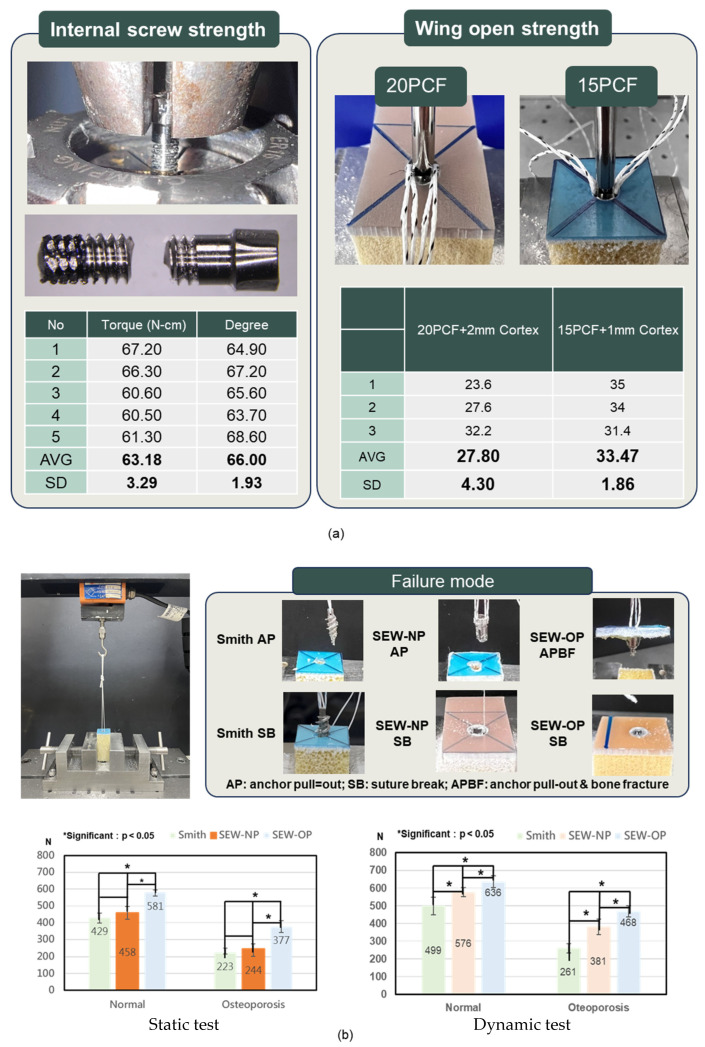
Mechanical performance of SEW anchors. (**a**) Internal screw torsional strength and wing-opening torque in polyurethane foam blocks (20 PCF with 2 mm cortex and 15 PCF with 1 mm cortex). The average torsional strength of the internal screw was 63.18 ± 3.29 N·cm, and the average rotation angle was 66.00° ± 1.93°. The average torque required for wing deployment was 27.80 ± 4.30 N·cm and 33.47 ± 1.86 N·cm in 20 PCF and 15 PCF blocks, respectively. (**b**) Static and dynamic pull-out tests of Smith, SEW-NP, and SEW-OP anchors under normal and osteoporotic bone conditions. SEW-OP demonstrated significantly higher pull-out strength compared with Smith and SEW-NP anchors in both conditions (*p* < 0.05). Representative failure modes are shown: suture break (SB) and anchor pull-out (AP) for Smith and SEW-NP, and bone block fracture (SB) for SEW-OP.

According to the ASTM F543 standard [40], mechanical performance tests for internal screws were conducted, including the “internal screw torsion strength test” and the “wing deployment test.” The tests were performed at a speed of 1080°/min with an axial preload of 10 N. In the torque strength test, the internal screw was placed in a fixed fixture and inserted along the thread until fully locked, then continuously rotated until the screw body fractured (Figure 4a). The maximum torque value (N·cm) and the fracture rotation angle (°) were recorded. In the wing deployment test, the anchor equipped with the internal screw and wing mechanism was fixed in polyurethane foam bone models of different densities (20 PCF + 2 mm cortical layer and 15 PCF + 1 mm cortical layer). The screw was rotated along its axis to trigger the wing deployment, and the torque value (N·cm) was recorded after setting a fixed number of deployment rotations based on bone density (Figure 4a).

The mechanical performance tests of the anchors in this study included static pull-out tests and pull-out tests after dynamic loading (Figure 4b), aiming to evaluate the primary stability and durability of the new anchors under different bone quality conditions. Artificial bone test blocks were prepared before the experiments, divided into normal and osteoporotic groups, and pre-drilled (5 mm) with no tapping and implanted according to the SEW-OP, SEW-NP, and Smith groups. In the SEW-OP group, after the anchor was implanted, the anchor was directly inserted until seated against the cortical surface. The proximal knob of the delivery handle was then tightened, which advances the internal screw and driven the expandable wings outward until their tips engage the cortical bone. Once the knob was fully secured, the deployment is complete, and the preloaded sutures can be passed and tied according to the reconstruction procedure. This protocol mirrors existing anchor workflows and requires no additional instrumentation beyond the dedicated inserter. The corresponding number of rotations for the normal and osteoporotic groups was 4.75 and 7.5 turns, respectively. Subsequently, the artificial bone blocks were clamped onto the testing machine for static pull-out tests, and the maximum pull-out strength and failure modes were recorded. Dynamic strength testing simulated the fatigue scenarios of daily shoulder joint activities with cyclic loading. A cyclic load of 15–150 N was applied at a frequency of 0.5 Hz for 300 cycles, followed by a pull-out test to observe the failure strength and mode of failure.

In summary, these tests simultaneously reflected the performance of the anchor in terms of initial stability, clinical load stability, and long-term tolerance, providing a comprehensive biomechanical validation basis.

### 2.5. Biomechanical Evaluation of SEWs at Coracoclavicular Reconstruction Model

**Figure 5:**
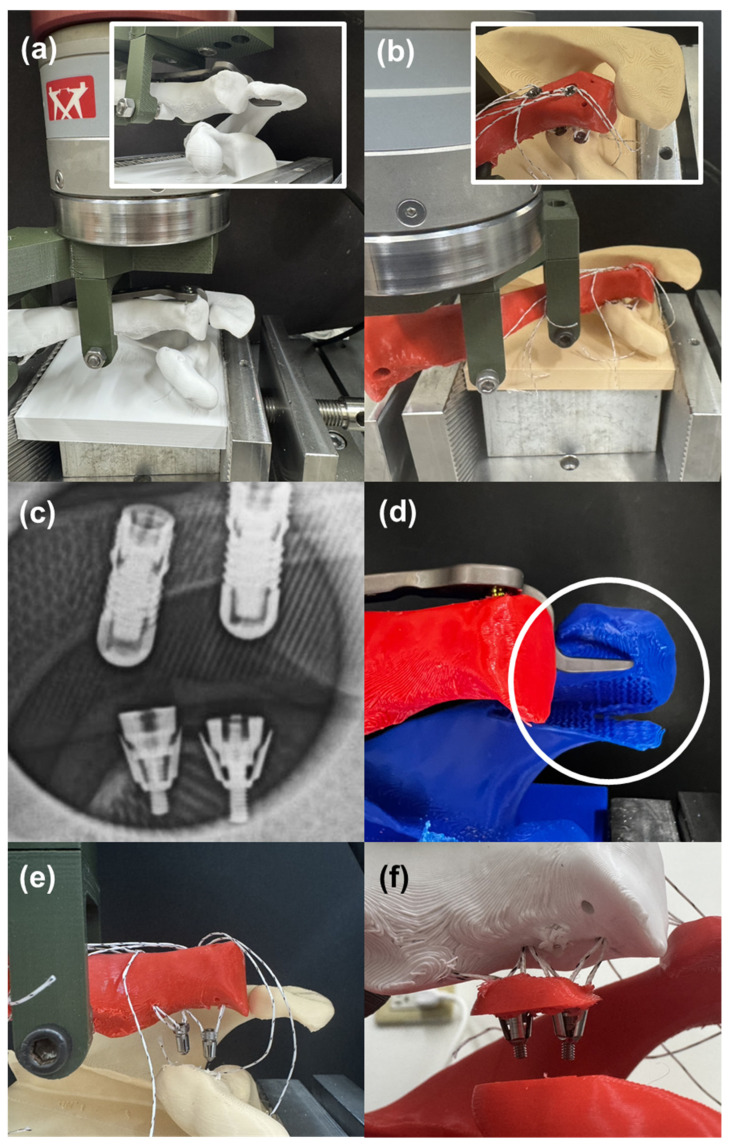
Coracoclavicular (CC) ligament reconstruction model and fixation outcomes. (**a**) Experimental setup using a traditional hook plate clasped beneath the acromion. (**b**) Fixation with the SEW anchor implanted into the coracoid process. (**c**) Radiograph confirming successful deployment of SEW anchor wings after implantation. (**d**) Hook plate fixation resulting in acromial fracture and structural disintegration. (**e**) SEW-NP anchors (wings not deployed) showing individual anchor pull-out failure. (**f**) SEW-OP anchors (wings deployed) resisting individual pull-out and instead failing together with the coracoid bone block, demonstrating superior fixation strength.

This study established a mechanical model for coracoclavicular (CC) ligament reconstruction, using CT imaging to reconstruct the anatomical structures of the scapula and clavicle. The distal end of the scapula was simplified into a single block model for experimental clamping, and a standardized model was created using 3D printing to ensure consistency between different test groups. There were three comparison groups: the first group was the traditional hook plate, which was fixed to the clavicle, with the hook end clasped to the bottom of the acromion to simulate the commonly used clinical method of fixing the acromioclavicular joint (Figure 5a). The second group was the new short-winged suture anchor with open wings (SEW-OP), which involved implanting two anchors into the coracoid process, threading the suture through the clavicle, and securing it with a suture-embedded anchor (Figure 5b,c). The third group was the new short-winged suture anchor with closed wings (SEW-NP), which was operated in the same manner as SEW-OP but kept the wings in a closed position.

Each experimental group was set with three samples (*N* = 3), totaling nine tests. All models were subjected to load testing using a tensile testing machine, with a custom clamp holding the clavicle and applying an upward force at a rate of 5 mm/min. The pull-out strength and failure modes of the clavicle and scapula were recorded to compare the fixation performance of the traditional plate and different anchor designs under high-demand clinical conditions.

## 3. Results

In a series of Sawbone compression tests, it was found that the elastic modulus of 20 PCF-solid and 15 PCF-cellular (131.82 MPa and 74.2 MPa) were closest to the elastic modulus values of normal bone (133.63 MPa) and severe osteoporosis (69.05 MPa) after conversion from the literature (Figure 2), with an error of less than 10%. Therefore, these two materials were used for FE simulation and as experimental artificial bone blocks.

**Figure 6:**
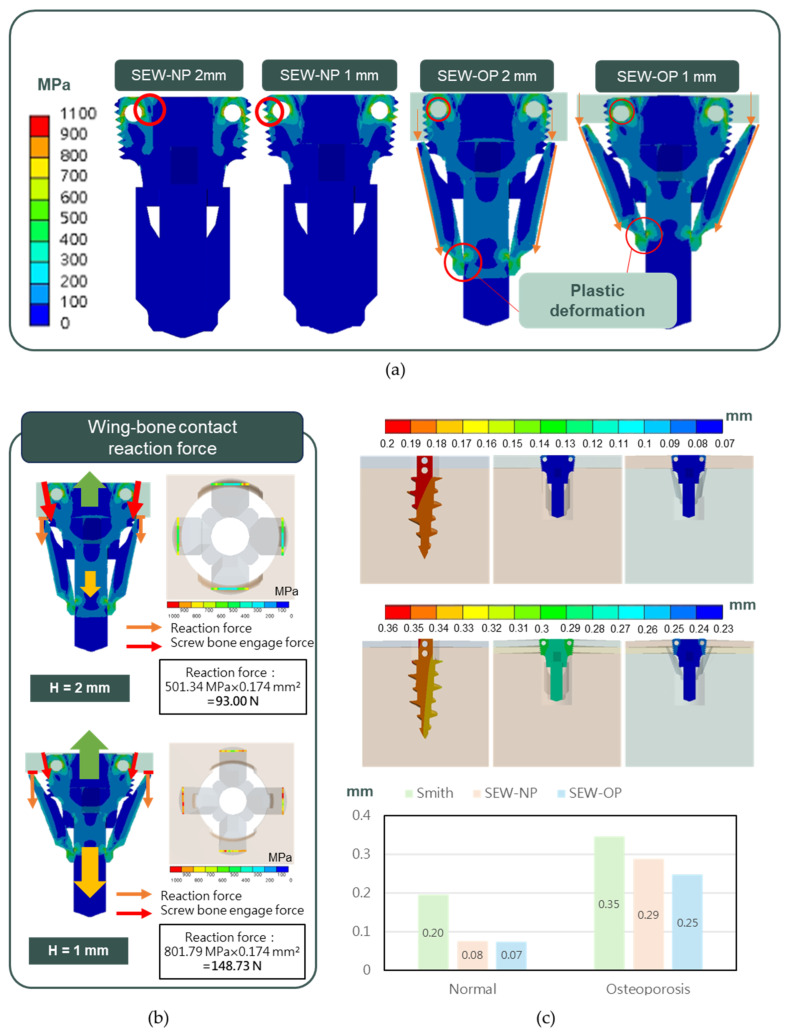
FE analysis of SEW and control anchors under normal and osteoporotic bone conditions. (**a**) Top: von Mises stress distribution showing concentration around the suture holes and bone–anchor contact areas, with plastic deformation occurring at the wing bottoms. (**b**) Bottom left: reaction forces at the wing–bone interface under cortical bone thicknesses of H = 2 mm (normal bone, 93.0 N) and H = 1 mm (osteoporotic bone, 148.7 N). (**c**) Bottom right: displacement patterns of Smith, SEW-NP, and SEW-OP anchors in normal and osteoporotic bone models. SEW-OP exhibited the lowest intra-bone displacement (0.07 mm in normal bone and 0.25 mm in osteoporotic bone), indicating enhanced initial fixation stability compared with Smith and SEW-NP anchors.

FE analysis showed that whether the wings were open or closed, stress concentration occurred at the force application locations, specifically around the suture holes and the areas in contact with the bone, but the values were below the fracture strength of titanium alloy. In the case of the anchor with the wings open, stress was generated at the bottom of the wings due to plastic deformation, and contact pressure was also generated at the tip of the wings due to impact with the cortical bone (Figure 6a). The reaction force generated by the contact pressure was 93.0 N under normal bone conditions, while under osteoporotic conditions it increased to 148.7 N, reflecting that local contact stress increased when bone quality decreased (Figure 6b). In terms of displacement (i.e., initial stability), under both normal bone conditions (H = 2 mm) and osteoporotic conditions (H = 1 mm), traditional Smith anchors exhibited greater intra-bone displacement (0.20 mm and 0.35 mm, respectively). The new type of anchor partially reduced displacement (0.08 mm and 0.29 mm) when the wings were not deployed (SEW-NP), and further significantly reduced displacement when the wings were deployed (SEW-OP), decreasing to 0.07 mm in normal bone and 0.25 mm in osteoporotic bone, indicating that the wing design effectively enhanced initial stability (Figure 6c).

**Figure 7:**
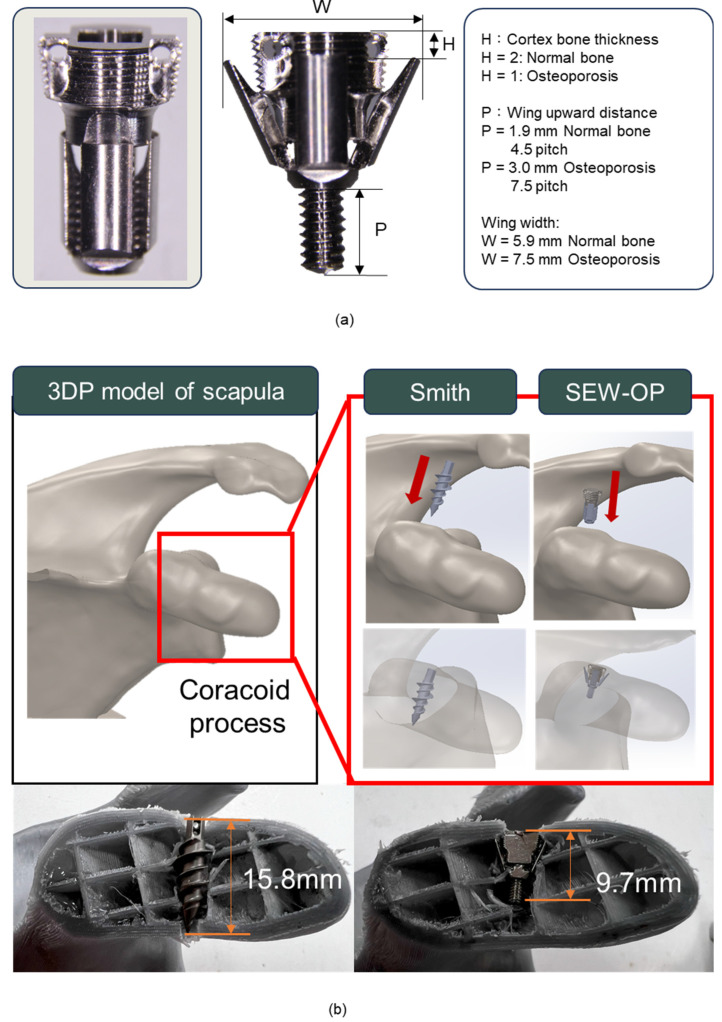
SEW anchor design parameters and implantation comparison in the coracoid process model. (**a**) Dimensional features of the SEW anchor, including cortical bone thickness (H) and wing elevation distance (P). Under normal bone conditions (H = 2 mm), P was approximately 4.5 pitches (3.0 mm), whereas under osteoporotic conditions (H = 1 mm), P increased to 7.5 pitches (1.9 mm). (**b**) 3D-printed scapula model showing anchor implantation. Compared with the Smith anchor, which required a lateral insertion path and an implantation depth of ~15.8 mm, the SEW-OP anchor was successfully implanted closer to the coracoid base with a reduced depth of ~9.7 mm, demonstrating suitability for limited bone volume.

The anchors and corresponding implantation instrument developed by our research institute were all manufactured by QMS-certified vendors, ensuring the high precision and operational stability required for clinical applications after processing. Especially at the suture holes on both sides of the anchor head, high-precision rounding and polishing treatments were performed to avoid suture wear or cutting during clinical operations (Figure 7a).

In terms of design parameters, H (cortical bone thickness) was inversely proportional to W (wing width) and P (wing pitch angle): when H = 2 (simulating normal bone), W and P were approximately 5.9 mm and 4.5 pitches (1.9 mm); when H = 1 (simulating osteoporotic bone), due to insufficient supporting bone thickness, P needed to be extended to about 7.5 pitches (3 mm), and W expanded to about 7.5 mm (Figure 7a). This design effectively simulated the winged state of the anchor under different bone conditions and verified its fixation performance. In the application model of the coracoid process, the 3D-printed scapula model showed that the traditional Smith anchor, due to its longer body, had a cross-sectional analysis that revealed that the actual implantation depth was approximately 15.8 mm, approximately penetrating the coracoid process. In contrast, the new winged anchor (SEW-OP), due to its shorter structure and winged fixation design, was successfully implanted closer to the base of the coracoid process, with the required implantation depth measured at only 9.7 mm (Figure 7b). These results indicated that the SEW-OP significantly reduced the required implantation depth in the limited bone volume area of the coracoid process, thereby decreasing the risk of fracture or penetration and enhancing its clinical applicability in small-sized bone structures.

The average torque strength of the internal screws was 63.18 ± 3.29 N·cm, with an average rotation angle of 66.00° ± 1.93°. The wing-opening torque was 27.80 ± 4.30 N·cm at 20 PCF with 2 mm cortex and 33.47 ± 1.86 N·cm at 15 PCF with 1 mm cortex, indicating that the torque required for SEW anchor wing opening was less than the torsional strength of the bone screws (Figure 4a). Static pull-out tests showed that the pull-out strengths of Smith, SEW-NP, and SEW-OP under normal bone conditions were 429, 458, and 581 N, respectively; under osteoporotic conditions, they were 223, 244, and 377 N, respectively (Figure 4b), with SEW-OP significantly higher than Smith and SEW-NP (*p* < 0.05). The pull-out test after dynamic loading showed that under normal bone conditions, the three groups increased to 499, 576, and 636 N, respectively; under osteoporotic conditions, they were 281, 381, and 468 N (Figure 4b). After dynamic testing, each group showed an increase compared to static testing (normal bone +9.5–25.8%; osteoporotic +24.1–56.1%), and SEW-OP remained the highest, significantly superior to the other two groups (*p* < 0.05). The failure modes indicated that regardless of dynamic or static testing or anchor type, the failure mode of normal bone was mostly suture break, while in osteoporotic conditions, most were anchor pullouts (Table 1, Table 2, Table 3 and Table 4 and failure models referred to Figure 4b).

X-rays verified that the wings of the SEW anchor successfully unfolded after implantation, providing intrabone expansion fixation (Figure 5c). Experimental observations of CC ligament reconstruction showed that the bone plate was prone to bone fracture and disintegration due to hooking onto the acromion (Figure 5d); when using SEW-NP (wings not deployed), the two anchors were easily pulled out individually, resulting in insufficient fixation (Figure 5e); when using SEW-OP (wings deployed), the anchors did not come out individually but instead broke together with the coracoid bone block, indicating that its fixation strength was significantly superior to traditional designs (Figure 5f). Further failure strength test data of the bone models also supported the above observations: the average strength of the traditional bone plate and SEW-NP were 185.33 N and 174.95 N, respectively, with little difference, whereas SEW-OP reached 391.00 N, nearly twice that of the first two groups (*p* < 0.05). Overall, SEW-OP not only demonstrated higher pull-out resistance in the experimental model but also avoided the risk of simple anchor loosening.

## 4. Discussion

In this study, a SEW anchor was designed and validated, which showed better fixation performance than the traditional design in FE analysis, static pull-out and dynamic load pull-out tests, and the coracoclavicular ligament reconstruction model. SEW anchor significantly improved pull-out strength in both normal and osteoporotic environments, and achieved stable fixation in small bone areas such as the coracoid process at an implantation depth of <10 mm, indicating that its design can simultaneously solve the two major clinical challenges of osteoporosis and limited bone mass.

Although suture anchors are widely used in rotator cuff repair and ligament reconstruction, they often loosen due to insufficient fixation strength under osteoporotic conditions. Ntalos et al. reported that the anchor pull-out rate in patients with osteoporosis can reach 22.8% [16]. The results of the pull-out test indicated that all specimens failed by SB, indicating that the cortical engagement was sufficiently strong such that the bone–implant interface was no longer the weakest link, and the suture construct became the limiting factor in normal bone. In osteoporotic bone, by contrast, nearly all specimens failed by APBF, reflecting the inability of the thinner cortical layer to provide sufficient purchase, such that the wings fractured the cortical margin during pull-out. Only one osteoporotic specimen failed by SB. These findings suggest that in normal bone, SB reflects a positive shift of the weakest link to the suture, whereas in osteoporotic bone, APBF highlights the challenge of reduced cortical thickness. Importantly, across both bone conditions, the SEW-OP anchor achieved significantly higher fixation strength than the commercial Smith anchor, which failed consistently by simple AP, underscoring the superior cortical engagement of the SEW design.

Some commercially available anchors with expandable designs (such as the POBLOK anchor) provide additional pull-out resistance by deploying distal wings into the cancellous bone. However, in osteoporotic bone, the expansion mechanism offers limited fixation due to the lack of effective interlocking. In contrast, the SEW anchor developed in this study engages directly with the cortical bone through barbed wings, achieving more reliable fixation in small-sized bones such as the coracoid process. Jia et al. also pointed out that the average width of the coracoid process is only 10–12 mm, which is more limited for elderly patients with osteoporosis [26]. The SEW anchor proposed in this study combined a short design (≤10 mm) with a wing-spreading mechanism, which provided reliable fixation even in limited bone mass, filling the gap in the design of existing commercially available anchors.

In addition, the all-suture anchor, which has been widely used in the market in recent years, shows advantages in small joints with limited bone mass or labrum repair through small drill holes (1.6–2.5 mm) and soft bag-like structures. Its advantage is that it causes little bone damage and is suitable for multiple-point implantation. However, its initial fixation is generally lower than that of ordinary anchors. However, their fixation strength relies heavily on cortical thickness, and they are particularly prone to tunnel widening and early loss of pull-out strength under cyclic loading in high-demand applications such as coracoclavicular ligament reconstruction. In ligament reconstruction or areas with thin bone cortex that bear large loads, it often fails due to bone tunnel expansion or bone fracture, and is not suitable for CC reconstruction and other regions that need to bear high tension [17]. In contrast, the SEW anchor is designed to lock directly against the cortical shell through its expandable wing mechanism, thereby maintaining fixation even in osteoporotic or small-dimension bones. In our study, the SEW anchor achieved pull-out strengths of 377–581 N (osteoporotic vs. normal bone), which are substantially higher than the typical values reported for ASAs (100–200 N in osteoporotic bone and ~200–400 N in normal bone) [5,16,25,26,27,28,29].

The reason for reconstructing CT images and using 3D printing to produce CC reconstruction bone models in this study was mainly to ensure a high degree of consistency in geometric morphology and material properties. The coracoid process and clavicle region vary significantly among individuals, and cadaver specimens are often limited by scarcity and individual variations, making it difficult to establish a reliable comparison benchmark; animal bones, on the other hand, differ greatly from humans in terms of geometric shape, and cannot truly simulate the clinical stress environment. In contrast, 3D-printed models can be directly reconstructed from images to obtain standardized shapes, and can be made using the same materials and processes, avoiding the impact of differences in bone quality or morphology on test results. This method not only improves the repeatability of the experiment but also makes the mechanical comparison between different groups more credible, providing a more rigorous basis for evaluating the performance of the new suture anchor in CC ligament reconstruction. In addition, it is worth noting that recent studies have highlighted the use of topology-optimized mechanical porous structures (TMPS) and bio-inspired lattice geometries to enhance load distribution and osseointegration in large bone implants [41,42]. While these strategies are highly effective for optimizing bulk implant architectures, their translation to small cortical-dominant sites such as the coracoid process is limited, where the primary challenge is not porosity optimization but achieving secure fixation in very restricted bone dimensions. In contrast, the SEW anchor was intentionally designed with a compact profile and deployable wing mechanism to maximize cortical purchase, directly addressing the unique fixation requirements of osteoporotic and small-dimension bones.

Hook plates have long been used for the treatment of acute acromioclavicular dislocation. Although they provide immediate fixation, the complication rate is high, including subacromial bone erosion, acromial pain, fracture, and residual subluxation. Liu et al. (2020) reported that the incidence of acromial osteolysis and fracture in hook plate patients without coracoclavicular (CC) augmentation was significantly higher than that in the augmentation group [31]; Shih et al. (2021) showed that even with CC suture anchors, hook plates were still difficult to avoid related complications [30]. The results of the CC reconstruction model in this study further confirmed that the fixation strength of SEW-OP was nearly twice that of the hook plate, and the structural problems across the acromion were avoided, showing its potential advantages in reducing invasiveness and complications.

The coracoclavicular ligament is a key structure for maintaining the stability of the acromioclavicular joint, but the bone mass at its anatomical location is limited, which is more challenging for elderly patients with osteoporosis. This study showed that SEW anchor only required 9.7 mm of implantation depth to achieve stable fixation, which greatly reduced the risk of penetration and fracture compared with traditional long screws (about 15.8 mm). Previous studies showed that hook plates combined with CC augmentation can improve short-term stability [31,32,33,34,35,36,37,38,43], but there are still problems of acromial compression and secondary removal. In contrast, SEW anchor is completely implanted into the coracoid process and does not involve the subacromial space, which may reduce related complications and the need for secondary surgery.

This study proposed an innovative SEW anchor design and performed biomechanical verification; however, there are still some limitations, First, the homogeneous polyurethane foam used as the bone substitute provides a standardized and reproducible environment for controlled comparisons; however, it does not replicate the heterogeneous microarchitecture and anisotropic mechanical properties of human cancellous bone. This simplification may have led to an overestimation of the anchor’s performance and may not fully reflect the complex failure mechanisms that occur in vivo. Second, the use of only 3D-printed models to simulate CC reconstruction, which cannot completely reproduce in vivo bone healing and soft tissue interaction. Further cadaveric or animal experiments are needed for verification. Third, the mechanical tests were limited to uniaxial tensile pull-out, whereas anchors in the clinical setting experience multidirectional forces including shear, toggle, and torsional loading. These additional loading conditions could alter both the failure mode and fixation strength. Future studies employing cadaveric or patient-specific bone models and incorporating off-axis and cyclic multidirectional loading protocols are warranted to provide a more realistic assessment of implant stability.

## 5. Conclusions

This study developed and validated a short expandable wing (SEW) suture anchor for fixation in osteoporotic and small-dimension bones. By combining a compact profile with a deployable wing mechanism, the anchor demonstrated clear clinical utility for coracoclavicular ligament reconstruction, where conventional devices are limited by insufficient cortical purchase and loosening. Biomechanical testing confirmed superior fixation: pull-out strengths reached 581 N in normal bone and 377 N in osteoporotic bone, with fixation further increasing by 25–56% after cyclic loading. In a 3D-printed CC reconstruction model, the SEW anchor provided nearly twice the fixation strength of the hook plate, underscoring its translational potential. The simple two-part design also suggests feasibility for industrial-scale manufacturing. Limitations include the use of artificial bone and uniaxial loading, warranting cadaveric studies, off-axis testing, and clinical trials. Collectively, the SEW anchor represents a promising next-generation fixation device for osteoporotic and small-bone applications.

## Figures and Tables

**Table 1:** Failure strength (N) and failure modes of anchors under static 0° pull-out in normal bone.

Anchor	Smith		SEW-NP		SEW-OP	
No.	Max Load	Failure Mode	Max Load	Failure Mode	Max Load	Failure Mode
1	455	AP	515	SB	560	SB
2	392	SB	448	SB	604	SB
3	435	AP	455	SB	574	SB
4	409	AP	406	AP	584	SB
5	457	AP	470	AP	582	SB
AVG	429		458		581	
SD	28.59		39.34		16.01	

AP: anchor pull = out; SB: suture break.

**Table 2:** Failure strength (N) and failure modes of anchors under static 0° pull-out in osteoporotic bone.

Anchor	Smith		SEW-NP		SEW-OP	
No.	Max Load	Failure Mode	Max Load	Failure Mode	Max Load	Failure Mode
1	212	AP	216	AP	363	APBF
2	202	AP	242	AP	383	APBF
3	197	AP	262	AP	348	APBF
4	248	AP	287	AP	358	APBF
5	257	AP	214	AP	436	APBF
AVG	223		244		377	
SD	27.4		31.07		35.46	

AP: anchor pull = out; APBF: anchor pull-out & bone fracture.

**Table 3:** Failure strength (N) and failure modes of anchors after dynamic loading in normal bone.

Anchor	Smith		EPACT UE		EPACT E	
No.	Max Load	Failure Mode	Max Load	Failure Mode	Max Load	Failure Mode
1	481	AP	543	SB	659	SB
2	585	AP	572	AP	592	SB
3	499	AP	586	SB	615	SB
4	456	AP	605	SB	636	SB
5	476	AP	582	SB	678	SB
AVG	499		576		636	
SD	50.33		26.32		34.13	

AP: anchor pull = out; SB: suture break.

**Table 4:** Failure strength (N) and failure modes of anchors after dynamic loading in osteoporotic bone.

Anchor	Smith		SEW-NP		SEW-OP	
No.	Max Load	Failure Mode	Max Load	Failure Mode	Max Load	Failure Mode
1	241	AP	415	AP	493	SB
2	238	AP	314	AP	471	APBF
3	263	AP	409	AP	491	APBF
4	303	AP	363	AP	417	APBF
5	258	AP	402	AP	470	APBF
AVG	261		381		468	
SD	26.13		42.37		30.64	

AP: anchor pull = out; SB: suture break; APBF: anchor pull-out & bone fracture.

## Data Availability

The original contributions presented in this report are included in the article. Further inquiries can be directed to the corresponding author.

## References

[1] Chaudhry S., Dehne K., Hussain F. (2019). A review of suture anchors. Orthop. Trauma.

[2] Braunstein V., Ockert B., Windolf M., Sprecher C.M., Mutschler W., Imhoff A., Postl L.K.L., Biberthaler P., Kirchhoff C. (2015). Increasing pullout strength of suture anchors in osteoporotic bone using augmentation—A cadaver study. Clin. Biomech..

[3] Horoz L., Hapa O., Barber F.A., Hüsemoğlu B., Özkan M., Havitçioğlu H. (2017). Suture anchor fixation in osteoporotic bone: A biomechanical study in an ovine model. Arthrosc. J. Arthrosc. Relat. Surg..

[4] Rosso C., Weber T., Dietschy A., de Wild M., Müller S. (2020). Three anchor concepts for rotator cuff repair in standardized physiological and osteoporotic bone: A biomechanical study. J. Shoulder Elb. Surg..

[5] Yang Y.S., Shih C.A., Fang C.J., Huang T.T., Hsu K.L., Kuan F.C., Su W.R., Hong C.K. (2023). Biomechanical comparison of different suture anchors used in rotator cuff repair surgery-all-suture anchors are equivalent to other suture anchors: A systematic review and network meta-analysis. J. Exp. Orthop..

[6] Cho C.H., Bae K.C., Kim D.H. (2021). Biomaterials used for suture anchors in orthopedic surgery. Clin. Orthop. Surg..

[7] Hsu W.C., Wu G.L., Yeh M.L. (2024). Fixation technique of biodegradable magnesium alloy suture anchor in the rotator cuff repair of the shoulder in a goat model: A technical note. BMC Musculoskelet. Disord..

[8] Chae S.W., Kang J.Y., Lee J., Han S.H., Kim S.Y. (2018). Effect of structural design on the pullout strength of suture anchors for rotator cuff repair. J. Orthop. Res..

[9] Trindade R., Albrektsson T., Galli S., Prgomet Z., Tengvall P., Wennerberg A. (2018). Bone immune response to materials, part I: Titanium, PEEK and copper in comparison to sham at 10 days in rabbit tibia. J. Clin. Med..

[10] Guyer R.D., Abitbol J.J., Ohnmeiss D.D., Yao C. (2016). Evaluating osseointegration into a deeply porous titanium scaffold: A biomechanical comparison with PEEK and allograft. Spine.

[11] Zhang M., Deng L., Zhang B., Liu J., Yang C., Liu T., Yang Z., Jiang J., Kang X., Yun X. (2025). Enhancing rotator cuff repair in rabbit osteoporosis with chitosan quaternary ammonium salt-coated nickel-titanium memory alloy anchors. Am. J. Sports Med..

[12] Chen C.H., Chang W.J., Chen Y.S., Chen K.H., Huang S.F., Hsueh H.R., Li C.B., Lin C.L. (2022). Development of a novel hybrid suture anchor for osteoporosis by integrating titanium 3D printing and traditional machining. Int. J. Bioprinting.

[13] Yamauchi S., Tsukada H., Sasaki E., Sasaki S., Kimura Y., Yamamoto Y., Tsuda E., Ishibashi Y. (2022). Biomechanical analysis of bioabsorbable suture anchors for rotator cuff repair using osteoporotic and normal bone models. J. Orthop. Sci..

[14] Schonebaum D.I., Garbaccio N., Escobar-Domingo M.J., Wood S., Smith J.E., Foster L., Mehdizadeh M., Cordero J.J., Foppiani J.A., Choudry U. (2025). Comparing biomechanical properties of bioabsorbable suture anchors: A comprehensive review. Biomimetics.

[15] Li X., Xiao Y., Shu H., Sun X., Nie M. (2022). Risk factors and corresponding management for suture anchor pullout during arthroscopic rotator cuff repair. J. Clin. Med..

[16] Ntalos D., Huber G., Sellenschloh K., Saito H., Püschel K., Morlock M.M., Frosch K.H., Klatte T.O. (2021). All-suture anchor pullout results in decreased bone damage and depends on cortical thickness. Knee Surg. Sports Traumatol. Arthrosc..

[17] Fat D.L., Kennedy J., Galvin R., O’Brien F., Grath F.M., Mullett H. (2012). The Hounsfield value for cortical bone geometry in the proximal humerus—An in vitro study. Skelet. Radiol..

[18] Morgan E.F., Bayraktar H.H., Keaveny T.M. (2003). Trabecular bone modulus-density relationships depend on anatomic site. J. Biomech..

[19] Liu G., Wang X., Wang X., Zhang Y., Ma Y., Zhou H., Shen G. (2025). Opportunistic screening for local osteoporosis of the proximal humerus on the basis of Hounsfield units: A study of patients with rotator cuff tears. Orthop. Surg..

[20] Reeves J.M., Knowles N.K., Athwal G.S., Johnson J.A. (2018). Methods for post hoc quantitative computed tomography bone density calibration: Phantom-only and regression. J. Biomech. Eng..

[21] Lin Y.S., Chang Y.Z., Yu J.H., Lin C.L. (2014). Do dual-thread orthodontic mini-implants improve bone/tissue mechanical retention?. Implant Dent..

[22] Weidling M., Heilemann M., Schoenfelder S., Heyde C.E. (2022). Influence of thread design on anchorage of pedicle screws in cancellous bone: An experimental and analytical analysis. Sci. Rep..

[23] de Kater E.P., Blom M.N., van Doorn T.C., Tieu Q.H., Jager D.J., Sakes A., Breedveld P. (2024). Enhancing spinal bone anchor pull-out resistance with an L-shaped anchor. PLoS ONE.

[24] Nagamoto H., Yamamoto N., Itoi E. (2018). Effect of anchor threads on the pullout strength: A biomechanical study. J. Orthop..

[25] Kim J.H., Hyeok J.J., Woo J.H., Kim S.M. (2025). Correlation analysis of suture anchor pull-out strength with cortical bone thickness and cancellous bone density on a finite element model. Bioengineering.

[26] Yu C., Sun L., Gao H., Sheng H., Feng X., Yang X., Li J., Kong Q., Hao Y., Feng S. (2024). Rotator cuff repair with all-suture anchor enhances biomechanical properties and tendon-bone integration in a rabbit model. Heliyon.

[27] Alt P.S., Marx C., Braun S. (2024). All-suture anchor size and drill angle influence load to failure in a porcine model of subpectoral biceps tenodesis, a biomechanical study. BMC Musculoskelet. Disord..

[28] Ock J., Seo J., Koh K.H., Kim N. (2023). Comparing the biomechanical properties of conventional suture and all-suture anchors using patient-specific and realistic osteoporotic and non-osteoporotic phantom using 3D printing. Sci. Rep..

[29] Ergün S., Akgün U., Barber F.A., Karahan M. (2020). The clinical and biomechanical performance of all-suture anchors: A systematic review. Arthrosc. Sports Med. Rehabilitation.

[30] Shih J.T., Yeh T.T., Wu C.C., Shen P.H., Wang C.C., Chien W.C., Chung C.H., Wang S.H. (2021). Hook plate fixation with and without coracoclavicular ligament augmentation with suture anchors for acute acromioclavicular joint dislocation. J. Med Sci..

[31] Liu C.T., Yang T.F. (2020). Hook plate with or without coracoclavicular ligament augmentation in the treatment of acute acromioclavicular separation. BMC Musculoskelet. Disord..

[32] Huang L., Cai L., Fan M., Yu P., Tu D. (2024). Subacromial osteolysis following hook plate fixation for acromioclavicular dislocation: A systematic review and meta-analysis. J. Shoulder Elb. Surg..

[33] Jia Y., He N., Liu J., Zhang G., Zhou J., Wu D., Wei B., Yun X. (2020). Morphometric analysis of the coracoid process and glenoid width: A 3D-CT study. J. Orthop. Surg. Res..

[34] Eecke E.V., Struelens B., Muermans S. (2024). Long-term clinical and radiographic outcomes of arthroscopic acromioclavicular stabilization for acute acromioclavicular joint dislocation. Clin. Shoulder Elb..

[35] Hong C.K., Kuan F.C., Hsu K.L., Chen Y., Chiang C.H., Su W.R. (2024). Biomechanical comparison of coracoclavicular fixation using metallic versus all-suture anchors. Orthop. J. Sports Med..

[36] Liu S., Li C., Song Z., Bai X., Wu H. (2022). Comparison of open reduction and fixation with hook plate and modified closed reduction and fixation with tightrope loop plate for treatment of rockwood type III acromioclavicular joint dislocation. BMC Musculoskelet. Disord..

[37] Shin S.J., Kim N.K. (2015). Complications after arthroscopic coracoclavicular reconstruction using a single adjustable-loop-length suspensory fixation device in acute acromioclavicular joint dislocation. Arthrosc. J. Arthrosc. Relat. Surg..

[38] Jeong J.Y., Chun Y.M. (2020). Treatment of acute high-grade acromioclavicular joint dislocation. Clin. Shoulder Elb..

[39] (2021). Standard Specification for Wrought Titanium-6Aluminum-4Vanadium ELI (Extra Low Interstitial) Alloy for Surgical Implant Applications.

[40] (2023). Standard Specification and Test Methods for Metallic Medical Bone Screws.

[41] Alemayehu D.B., Todoh M., Huang S.J. (2025). Hybrid biomechanical design of dental implants: Integrating solid and gyroid triply periodic minimal surface lattice architectures for optimized stress distribution. J. Funct. Biomater..

[42] Zhang J., Chen X., Sun Y., Yang J., Chen R., Xiong Y., Hou W., Bai L. (2022). Design of a biomimetic graded TPMS scaffold with quantitatively adjustable pore size. Mater. Des..

[43] Liu Y., Zhang X., Yu Y., Ding W., Gao Y., Wang Y., Yang R., Dhawan V. (2021). Suture augmentation of acromioclavicular and coracoclavicular ligament reconstruction for acute acromioclavicular dislocation. Medicine.

